# Hyperthyroidism With Atrial Fibrillation in Children: A Case Report and Review of the Literature

**DOI:** 10.3389/fendo.2021.689497

**Published:** 2021-09-20

**Authors:** Deepa Subramonian, Yuwei Juliana Wu, Shazhan Amed, Shubhayan Sanatani

**Affiliations:** ^1^Division of Pediatric Cardiology, University of British Columbia, Vancouver, BC, Canada; ^2^Division of Pediatric Endocrinology, University of British Columbia, Vancouver, BC, Canada

**Keywords:** atrial fibrillation, hyperthyroidism, heart, thyroid hormones, arrhythmia, cardioversion

## Abstract

Atrial fibrillation is exceedingly rare in children with structurally and functionally normal hearts. We present a novel case of a 15-year-old female with known hyperthyroidism who subsequently developed atrial fibrillation. She had been suffering from fatigue, heat intolerance and myalgias for 6 months. Her initial TSH was 0.01mU/L, and free T4 was 75.4 pmol/L, with a free T3 of >30.8 pmol/L. An electrocardiogram showed atrial fibrillation with a ventricular rate of 141 beats per minute. An echocardiogram demonstrated an enlarged left atrium and ventricle, with mild mitral regurgitation. She was treated with methimazole and underwent synchronized cardioversion. She subsequently returned to a euthyroid state and remained in normal sinus rhythm. In this case, we discuss the physiologic and arrhythmogenic properties of thyroid hormone, with a summary of the existing literature on atrial fibrillation in hyperthyroidism in children. Current guidelines for treatment of atrial fibrillation are also outlined.

## Introduction

Atrial fibrillation (AF) occurs commonly in hyperthyroidism in the adult population. However, it is a rare occurrence in children (1). To date, there have been three reported cases of hyperthyroidism accompanied by AF in children (2–4), and one reported case of atrial flutter associated with hyperthyroidism (5). We report a rare case of a 15-year-old female with hyperthyroidism complicated by AF. The important link between endocrine disorders and the heart is discussed, along with the physiologic and arrhythmogenic properties of thyroid hormone. A summary of current guidelines for the treatment of AF in hyperthyroidism in children is also provided.

### Case Report

A 15-year-old female was referred to the cardiology clinic with tachycardia, murmur and known hyperthyroidism. She had been suffering from fatigue, heat intolerance and nonspecific myalgias for six months, and subsequently developed shortness of breath on exertion. Her past medical history was unremarkable and there was no known family history of AF.

On initial visit, her weight was 53.9 kg (57.6^th^ percentile, +0.19 SDs) and height was 158.8 cm (31.9^th^ percentile, -0.47 SDs). Heart rate was 148 beats per minute and irregular, with a blood pressure of 138/72 mmHg. The precordium was hyperdynamic with normal first and second heart sounds, and a gallop rhythm. There was a grade II/VI systolic regurgitant murmur audible at the left lower sternal border and over the apex. Her thyroid was moderately enlarged with no palpable nodules. There was no peripheral edema or hepatosplenomegaly.

#### Investigations

**Figure 1:**
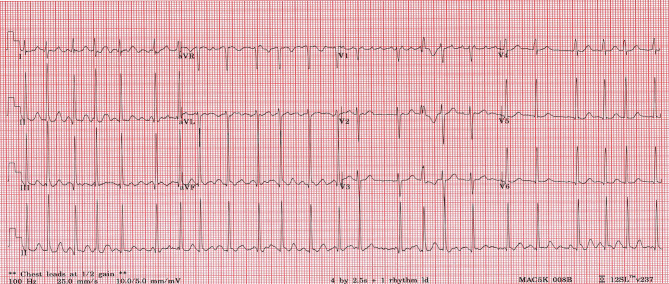
12 lead ECG demonstrating AF with variable conduction and left ventricular hypertrophy.

An electrocardiogram (ECG) showed AF with an average ventricular rate of 141 beats per minute, with irregular conduction and left ventricular hypertrophy (**Figure 1**). Laboratory studies revealed a TSH of 0.01mU/L, free T4 of 75.4 pmol/L, free T3 of >30.8 pmol/L, thyroperoxidase antibodies of 114 IU/mL, and TSH receptor antibody of 25 U/L. She had a normal complete blood count, electrolytes and renal function (**Table 1**). An echocardiogram revealed an enlarged left atrium, left ventricle (>6cm), moderate mitral regurgitation and mild mitral valve prolapse with low normal cardiac function (**Table 2**).

**Table 1:** Lab values.

Lab values	Pre-treatment	5months post-treatment	Reference range
Free T4 (pmol/L)	75.4	16.7	10.5 - 20
Free T3 (pmol/L)	>30.8	6.7	3.5 - 6.5
TSH (mU/L)	0.01	<0.06	0.3 - 6.0
Thyroperoxidase antibody (IU/ml)	114		0 - 9
TSH receptor antibody(U/L)	25		< 1.8
Hematocrit	0.391		0.350 - 0.440
Sodium (mmol/L)	143		135 - 145
Potassium (mmol/L)	4.1		3.5 - 5.0
Creatinine (umol/L)	38		39 - 97

T4, Thyroxine; T3, Triiodothyronine; TSH, Thyroid stimulating hormone.

Thyroid function was tested frequently after initiation of methimazole until stabilization. This thyroid panel represents 5 months after presentation when thyroid function stabilized.

**Table 2:** Key Echocardiogram parameters.

Parameters	Value	Z score
LV end-diastolic dimension	6.1 cm	3.18
LV end-systolic dimension	4.2 cm	3.25
Interventricular septum thickness in diastole	1.0 cm	1.37
LV posterior wall thickness in systole	1.2 cm	0.31
LV posterior wall thickness in diastole	0.6 cm	0.49
LV ejection fraction	59%	
LV fractional shortening	31.9%	
LV wall stress	128.3 g/cm^2^	
LV Cardiac Index	2.73 l/min/m^2^	
Left atrial diameter	4.9 cm	
Mitral Valve E/A	1.0	
Mitral Valve E Velocity	1.3 m/s	

LV, Left Ventricle; E/A, Ratio of E (early diastolic wave)/A (late diastolic wave); E Velocity, early diastolic velocity.

#### Treatment and Follow Up

**Figure 2:**
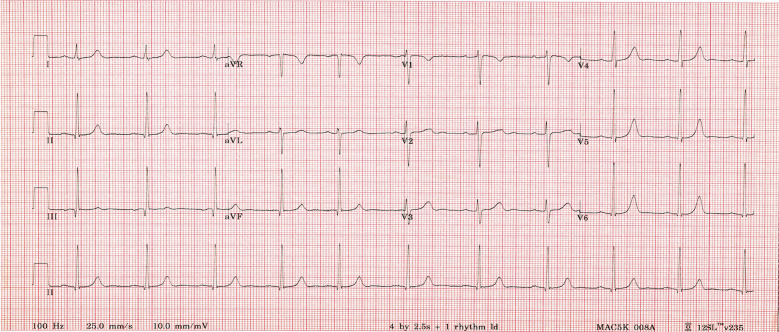
12 lead ECG demonstrating sinus rhythm.

The patient had a transesophageal echocardiogram that demonstrated no thrombus in the left atrium. She underwent synchronized cardioversion as daycare procedure, and successfully reverted to normal sinus rhythm at a rate of 90 beats per minute (**Figure 2**). Warfarin was started post cardioversion and anticoagulation was discontinued after evidence of persistent sinus rhythm. Low dose aspirin was added as part of our institutional practice to use aspirin for patients with AF at low risk of an embolic event. Her medical management included methimazole at a dose of 0.5 mg/kg/day and bisoprolol 2.5mg once daily. A euthyroid state was achieved, and AF did not recur. She was seen in follow-up clinic in a week, a month, 6 months and 1 year period. She continued to be in sinus rhythm. Bisoprolol was discontinued 4 months after her initial presentation.

## Discussion

The incidence of AF is rare in childhood, and the recognized association of adult thyroid disease and atrial fibrillation does not occur in pediatrics (1). In hyperthyroidism, the incidence of AF was found to be high in those with male sex, advancing age, coronary heart disease, congestive heart failure and valvular heart disease. Even subclinical hyperthyroidism is associated with a threefold increase in the risk of atrial fibrillation in older persons (6). Hyperthyroid patients may experience more significant clinical symptoms such as palpitations, fatigue, dizziness, reduced exercise capacity or mild dyspnea when accompanied by AF. We present a rare case of AF in a 15-year-old female with known hyperthyroidism. It is important to note the link between endocrine disorders and the heart. Thyroid hormones have several effects on the cardiovascular system (7–9).

The thyroid secretes two hormones, thyroxine (T4), a prohormone, and triiodothyronine (T3), the active hormone (7). Thyroid hormone has both physiologic and arrhythmogenic properties (8, 10). In hyperthyroidism there is excessive production of T3 as a result of hypersecretion by the thyroid gland, and an increase in the peripheral monodeiodination of T4. This leads to marked changes in the cardiovascular system through both nuclear and nonnuclear actions at the cellular level (8, 11). T3 increases myocardial contractility, resting heart rate, systolic and mean pulmonary artery pressure, myocardial oxygen consumption and reduces afterload by acting on vascular smooth muscle and reducing systemic vascular resistance (8, 12, 13). Most of the measures of cardiac function, including left ventricular (LV) ejection fraction, the rate of ventricular pressure development, diastolic relaxation, and cardiac output, are increased (14). These functional changes are most likely the result of an increase in expression of myocardial sarcoplasmic reticulum calcium-dependent adenosine triphosphatase, a decrease in the expression of its inhibitor, phospholamban, and reduction in systemic vascular resistance (12, 13).

On the other hand, thyroid hormone has arrhythmogenic properties affecting both atria and ventricles (10). Elevated thyroid hormone (T3) results in increased sympathetic function, tachycardia and decreased atrial refractory period which are mediated by alteration in the sensitivity of β1-adrenergic and M2-muscarinic receptors of the heart, vagolytic effects and alteration of ionic channel activity by T3 respectively. Thyroid hormone decreases L-type calcium channel mRNA expression and increases expression of Kv 1.5 mRNA which causes increase in the rate of K efflux and in turn shortening of atrial refractory period (6). The effective refractory period shortening of atrial myocytes increases the susceptibility of cardiomyocytes to reactivation from electrical impulses, which in turn can lead to reentry atrial circuits and AF (10, 15, 16). Thyroid hormone also increases the automaticity of pulmonary vein cardiomyocytes (17). Pulmonary veins have been demonstrated to be important origins of ectopic beats in the initiation of AF (17, 18).

The physiology behind the initiation and maintenance of paroxysmal AF is multi-factorial. Dominant theories of the pathogenesis of AF highlight electrical remodeling, structural remodeling and inflammation (19). Hyperthyroidism is primarily known to affect electrical remodeling through shortening of the effective refractory period of atrial myocytes and in increasing the arrhythmogenicity of pulmonary vein myocytes.

Hyperthyroid patients with undetectable structural heart disease have more premature supraventricular depolarizations, and non-sustained supraventricular tachycardias, as well as premature atrial complex and reduced heart rate variability (20). The latter is primarily the result of decreased parasympathetic tone. These electrical triggers may contribute to paroxysmal atrial tachycardia, AF, and atrial flutter. Among these arrhythmias, AF is the most common (21). Mendelian randomization studies (22, 23) have provided evidence that the relationship between thyroid function and AF is causal.

Lone AF occurring in younger patients without underlying cardiovascular disease or comorbidities represents less than 5% of all types of AF (24, 25). The majority of documented AF in children has been associated with cardiomyopathy and congenital heart disease, such as transposition of great vessels (25). It has also been documented in children after undergoing cardiac surgery, namely the Fontan or Mustard procedures (25–27). There was an increased rate of AF in pediatric patients with unrepaired cardiac lesions resulting in significant left sided hemodynamic effects and in those who had never undergone cardiac surgery (26). The workup for AF should evaluate the presence of triggers, predisposing factors (family history, obesity, excessive participation in endurance sports, hyperthyroidism, smoking, alcohol use, drugs or stimulants use) and inherited channelopathies (28). Lone AF can occur with rare channelopathies in children like Brugada syndrome (BrS), Long QT syndrome (LQTS) and Short QT syndrome (SQTS). The prevalence of AF is 10-20%, 2-30% and upto 30% in patients with BrS, LQTS and SQTS respectively (28–30). Lone AF does carry substantial symptomatic burden and has a high recurrence rate (31). Identification of associated diseases considerably alters prognosis and therapy. However, we demonstrate a case of AF in a child with undetected structural heart disease, secondary to hyperthyroidism.

The specific management strategies for pediatric AF in general have not been well defined, but there is a need for an organized and consistent approach. Current guidelines for treatment of AF with hyperthyroidism recommend achieving a clinically euthyroid state, and most patients will spontaneously revert to sinus rhythm. Beta blockers are recommended to control the ventricular rate (32, 33). If there is persistent AF after return to euthyroid state, cardioversion is recommended (32–34). Adult studies have shown that a fraction of patients will remain in AF despite restoration of euthyroid state and may require catheter ablation (35). Recent adult studies also propose patients with hyperthyroidism may have an increased prevalence of pulmonary venous and non-pulmonary venous ectopic foci and are susceptible to an increased risk of recurrence of AF after a single ablation procedure (35).

Complications of AF in patients with hyperthyroidism include heart failure and thromboembolism, although it remains controversial whether AF in hyperthyroidism is associated with a higher thromboembolic risk than AF in other settings (13, 14, 36). Approximately 10 to 15 percent of adult patients with overt hyperthyroidism who have AF have an arterial embolic event (37, 38). The literature to date does not provide consistent recommendations on the need for anticoagulation in hyperthyroid patients with AF, with no published studies focusing on anticoagulants in childhood. The CHADS2 [Congestive Heart failure, Hypertension, Age ≥ 75, Diabetes, and prior Stroke or TIA (double)] or CHA2DS2- VASc [Congestive Heart failure, Hypertension, Age ≥ 75 (double), Diabetes, and prior Stroke or TIA (double), Vascular disease, Age 65–74, and Sex (female)] risk score is recommended to guide long-term anticoagulation decisions in adult patients (39, 40). While no such score has been validated in children, and subclinical stroke can occur early in the clinical course (41), even with a CHADS2 score of 0-1, the benefits of anticoagulant therapy have yet to be assessed. Like in adults, the CHA2DS2-VASc could be used to assess the stroke risk and in the absence of any risk factor (i.e., hypertension, diabetes, stroke, or TIA or vascular disease) oral anticoagulation is not recommended for young patients with lone AF. If duration of AF is more than 48 h or unknown, transoesophageal echocardiography or 4-weeks of anticoagulation therapy is recommended prior to cardioversion with anticoagulation continuing for 4 weeks following the cardioversion (39). Anticoagulation treatment should be continued for 4 weeks following the cardioversion regardless of the risk of thromboembolism (39). The anticoagulant therapy can be discontinued after successful treatment of the disorder along with periodic assessment for any recurrence of AF and guided by patient’s underlying risk for stroke (42). The absence of signs and symptoms of AF and 24 hr monitoring showing no AF can be considered adequate to stop anticoagulants.

The studies to date on AF in the young have required multicenter collaborations to gather reasonable numbers of patients (31, 43). It is not likely feasible to have large populations with AF and thyroid disease in the young, thus the novelty of this case. There are other methods to advance our knowledge. Since the last decade, preclinical studies involving human induced pluripotent stem cells derived cardiomyocytes have shown promising role in modelling various cardiac diseases including AF and as a potential platform for drug development (44, 45). In Hyperthyroidism with AF in children, given the paucity of studies and patient population, future research with human induced pluripotent stem cells-derived cardiomyocytes or tissue engineering are plausible tools to provide important insight into the mechanism of AF in hyperthyroidism and to develop specific management strategies.

This particular case is demonstrative of a rare and serious complication of hyperthyroidism in a child, and we have reviewed the physiology underlying the propensity toward AF in hyperthyroidism. The general principles in the treatment of AF secondary to hyperthyroidism have also been outlined. In the adult population, it is common for clinicians to investigate for thyroid disease in patients with AF. Thus, for the general pediatric clinician, it is important to have AF and atrial arrhythmias on the differential diagnosis of complications for the hyperthyroid patient.

Learning Points:

-There is an important link between endocrine disorders and the cardiovascular system.
-Thyroid hormone has both physiologic and arrhythmogenic properties.
-Patients with clinical hyperthyroidism have an increased risk of AF.
-Although rare, pediatricians should be aware of AF as a complication of hyperthyroidism in children with structurally and functionally normal hearts.

## Author Contributions

DS and YW prepared the body of the manuscript. SS and SA critically reviewed the publication. All authors contributed to the article and approved the submitted version.

## Conflict of Interest

The authors declare that the research was conducted in the absence of any commercial or financial relationships that could be construed as a potential conflict of interest.

## Publisher’s Note

All claims expressed in this article are solely those of the authors and do not necessarily represent those of their affiliated organizations, or those of the publisher, the editors and the reviewers. Any product that may be evaluated in this article, or claim that may be made by its manufacturer, is not guaranteed or endorsed by the publisher.

## Abbreviations

AF, Atrial fibrillation; ECG, electrocardiogram; FT4, free thyroxine; FT3, free triiodothyronine; LV, left ventricle; TSH, Thyroid stimulating hormone; T4, Thyroxine; T3, Triiodothyronine; TIA, Transient ischaemic attack.
